# Accuracy of a portable breath meter test for the detection of halitosis in children and adolescents

**DOI:** 10.6061/clinics/2020/e1764

**Published:** 2020-09-03

**Authors:** Carolina Cardoso Guedes, Sandra Kalil Bussadori, Ana Carolina Mota Garcia, Lara Jansiski Motta, Andréa Oliver Gomes, Raimar Weber, Olga Maria Silverio Amancio

**Affiliations:** I Departamento de Pediatria, Universidade Federal de Sao Paulo, Sao Paulo, SP, BR.; II Universidade Nove de Julho, São Paulo, SP, BR.; III Departamento de Otorrinolaringologia, Hospital Infantil Sabara, Sao Paulo, SP, BR.

**Keywords:** Halitosis, Diagnosis, Pediatric Dental Care

## Abstract

**OBJECTIVES:**

This study aimed to determine the accuracy of the Breath-Alert™ portable breath meter (BA) for the detection of halitosis in children and adolescents, considering the organoleptic test (OT) as the gold standard in this assessment.

**METHODS:**

A cross-sectional study was conducted on 150 children (aged 6-12 years). OT was performed by three independent examiners on a single occasion, obtaining three scores of 0-5 points on the Rosenberg’s organoleptic scale. The median of the three evaluations for each child was used for analysis. BA was used according to the manufacturer’s instructions, with breath odor scored from 0-5 points. Scores ≥2 on both tests were considered indicative of halitosis.

**RESULTS:**

A total of 26 (17.3%) and 23 (15.3%) children were detected with halitosis on the OT and BA tests, respectively. The sensitivity and specificity of the BA scores for the detection of halitosis were 80.76% and 98.38%, respectively. The positive and negative predictive values for BA were 91.3% and 96.06%, respectively.

**CONCLUSION:**

In the present study involving children, who require fast, practical examinations, BA proved to be an auxiliary tool to OT for the detection of halitosis in the practice of pediatric dentistry, demonstrating high sensitivity and specificity.

## INTRODUCTION

Halitosis is an imbalance in which breath odor is altered in an unpleasant manner, causing a significant social and psychological disadvantage for affected individuals (1). Globally, the prevalence of halitosis in the adult population ranges from 22-50% (2-5). Most studies on halitosis that have been conducted on children considered the opinions of parents (1,6); therefore, the actual prevalence of this condition remains unclear. Poor oral hygiene is associated with halitosis in the adult population and studies indicate that the same is true for pediatric populations as well (6,7).

The foul odor in exhaled air is caused by volatile sulfur compounds (VSCs) produced by anaerobic Gram-negative bacteria (8) on substrates rich in sulfur-containing amino acids (1,2,9-11). The concentration of these gases (sulfide, methanethiol, and dimethylsulfide) is used for the diagnosis of halitosis (12).

The literature describes two main methods for the assessment of halitosis: subjective (organoleptic) and objective (gas chromatography or a sulfide monitor) (1,13). The organoleptic test (OT) is a simple, low cost method that is considered the gold standard for assessment (14-16), in which a trained examiner uses his/her sense of smell to detect and classify bad breath. As this test depends on the examiner’s subjective judgment, standardization among studies is difficult (14-16). To overcome the limitations of OT, objective methods have been developed to measure halitosis, such as the OralChroma™ gas chromatograph (Abilit Corporation, Miyamae-KU Kawasaki-shi, Kanagawa, Japan), Breath-Alert™ (Tanita Corporation, Japan), and Halimeter™ (Interscan Corporation, Chatsworth, CA, USA) portable sulfide monitors.

The Breath-Alert™ portable device (BA) has been increasingly employed in clinical practice due to its ease of use and low cost (14-19). This device measures VSCs and hydrocarbon gas, but few studies have verified its precision in clinical practice.

As a proper diagnosis is of the utmost importance to determine effective solutions for the treatment of halitosis (16), it is essential to establish effective, low-cost tests that can be easily performed on children for the diagnosis of halitosis in clinical practice. Thus, this study aimed to determine the accuracy of BA for the detection of halitosis in children in comparison to OT.

## METHODS

This study received approval from the Human Research Ethics Committee of the Federal University of São Paulo (certificate number: 610.481). All parents/guardians and children received clarifications regarding the objectives and procedures of the study and signed statements of informed consent.

A prospective cross-sectional study was conducted with children scheduled for dental treatment at the pediatric clinic of the pediatric dentistry specialization course of the Paulista Association of Dentists (São Paulo, Brazil). Children aged 6-12 years in the mixed dentition phase (primary and permanent teeth in the oral cavity) were included in the study. The exclusion criteria were tonsillitis, sinusitis, neurological, psychiatric, or behavioral disorders, and the use of medication. A total of 167 children were examined, and 150 of them who met the eligibility criteria were included in the study.

The evaluations were performed in two sessions. In the first session, the children and caregivers answered a questionnaire addressing personal data, general health, oral health, and lifestyle and eating habits. The children and caregivers were then instructed to avoid eating spicy and/or aromatized food 24 hours prior to the session, and eat an adequate meal 3 hours prior to the session, followed by habitual tooth brushing and flossing, without the use of mouthwash, breath mints, or chewing gum, prior to the second session. Children were also instructed not to use deodorants, perfumes, cosmetics, and creams that could &quot;confuse&quot; the judges.

In the second session, the children were clinically evaluated for halitosis using OT and BA.

### Organoleptic test

To minimize subjectivity, OT was performed by three different examiners in the same session, with each examiner blinded to the other assessments. Thus, each child was evaluated three times independently, obtaining three scores ranging from 0-5 points on the Rosenberg’s organoleptic scale (Table 1) (20-22). The median of the three scores was used for analysis. During the test, the examiner was positioned 10 cm from the patient. This distance from the child’s labiomental sulcus to the examiner’s nostrils was standardized using a disposable acetate ruler. Each child was instructed to exhale in the examiner’s direction and the examiner rated the breath odor emanating from the oral cavity (20,21). A score ≥2 points was considered indicative of halitosis.

### Breath-Alert™ test

**Figure 1:**
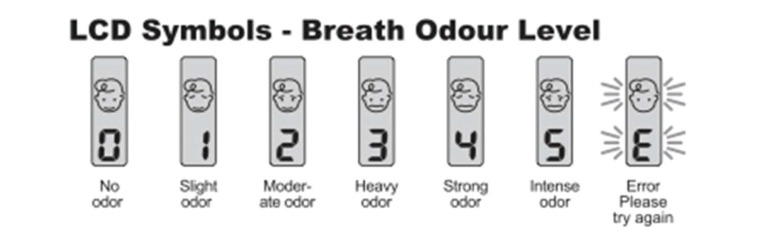
Halitosis scoring using a Breath-Alert™ device.

BA was used according to the manufacturer’s instructions and disinfected after each use. The device was shaken four or five times prior to each use to eliminate any residual odors. A “beep” was emitted on opening the upper compartment of the device, and a second “beep” was emitted when the volunteer blew into the frontal air entrance (air flow passage). After a third “beep,” the breath odor was measured and scored on a scale of 0-5 points. When the letter “E” appeared, indicating an error, the procedure was repeated (Figure 1). A score ≥2 was considered indicative of halitosis.

### Statistical analysis

The sample size was calculated considering the 52% prevalence rate reported by the Brazilian Halitosis Association, a 5% significance level, and 8% margin of error, leading to a minimum sample of 150 children. Sensitivity, specificity, positive predictive value (PPV), and negative predictive value (NPV) were calculated for the BA test in comparison to OT. Data analysis was performed using IBM SPSS (Subscription version 05-2017) for the MAC (Apple Inc.) operating system.

## RESULTS

The sample was composed of 150 children (54% girls and 46% boys) with a mean age of 9.1±1.4 years (range: 6.1-12.2 years). Twenty-six children (17.3%) were classified as having halitosis when assessed by OT. However, when assessed using BA, 23 (15.3%) were classified as having halitosis and 2 (1.3%) were false positives.

Table 2 displays the prevalence of halitosis using OT and BA. The sensitivity, specificity, PPV, and NPV of BA for the diagnosis of halitosis were 80.76%, 98.38%, 91.3%, and 96.06%, respectively (Table 3).

## DISCUSSION

OT is widely used since it is inexpensive, easy to administer and does not require any technological device (1,17,22). The disadvantage of this method is that it depends on the subjective judgment of the examiner (14,23), who needs to be trained, hindering the standardization and reproduction of OT in clinical practice and research. The ideal protocol involves the opinion of at least three independent examiners for the same patient and the determination of the median score, which improves the quality of the exam. For adults, this type of measurement is fast and easy. However, the test is more difficult for children due to the inherent immaturity of age, which makes the three repetitions tiring. Moreover, it is practically impossible to have three trained examiners available in routine clinical practice, which underscores the need for an objective exam. OT can also be considered embarrassing for both the examiner and patient, as the patient needs to exhale in the direction of the examiner’s nose at a short distance (10 cm) which is often considered to be very close and uncomfortable (14,15,24).

Alternatives to OT include the OralChroma™ gas chromatograph, Halimeter™, and Breath-Alert™. Gas chromatography is the most effective objective method to compare the efficacy of halitosis tests (20,25), as it is capable of measuring VSCs and the intensity and origin of halitosis. However, the cost of the device is too high for the purposes of clinical practice. The Halimeter™ measures the quantity of VSCs emitted through bad breath and determines the total in parts per billion (ppb). This device detects sulfide gas and methanethiol, but is not sensitive to dimethylsulfide (12,15). While its cost is moderate, its use for pediatric patients is challenging. BA has been increasingly used in clinical practice due to its ease of use, portable size, and low cost (14-18,26). This device measures VSCs and hydrocarbon gas, providing results that can signify a patient’s halitosis, but few studies have verified its precision in clinical practice (15).

In the present study, BA demonstrated high sensitivity (80.76%), although it failed to diagnose some children with halitosis that were positive on OT. This divergence may be because VSCs are the main contributors to bad breath, but other organic compounds are also found in exhaled air (15). Portable monitors exhibit variability and limitations in the identification of compounds and it is not yet possible to use such devices alone for the detection of halitosis (15), since the human nose is capable of detecting other organic compounds and define them as either pleasant or unpleasant (14,15,21,22). Therefore, OT continues to be considered the gold standard, despite being a subjective test.

To improve the reliability of diagnosis, halitosis should be assessed using two different methods (14), with OT as the subjective method of choice. The concomitant use of OT and BA could improve the diagnosis of halitosis, enabling a simple, fast, and reliable detection in the practice of pediatric dentistry. However, further studies are needed to evaluate each score separately and determine their accuracy in the different categories of these tests, and not just in the presence or not of halitosis.

## CONCLUSION

In the present study, BA was found to be useful for the rapid detection of halitosis in daily pediatric dental practice, demonstrating high sensitivity and specificity.

## AUTHOR CONTRIBUTIONS

Guedes CC was responsible for the conceptualization, data curation, investigation, methodology, visualization, and manuscript writing. Bussadori SK was responsible for the investigation, methodology, and visualization. Garcia ACM was responsible for the data curation, investigation, and visualization. Motta LJ was responsible for the formal analysis, software resources, and visualization. Gomes AO was responsible for the software resources and visualization. Weber R was responsible for the formal analysis and software resources. Amancio OMS was responsible for the conceptualization, data curation, methodology, and manuscript writing.

## Figures and Tables

**Table 1:** Rosenberg’s organoleptic scale (21).

0	No perceivable odor
1	Weakly perceivable odor
2	Perceivable odor
3	Moderate odor
4	Strong odor
5	Extremely strong odor

**Table 2:** Cross tabulation of the prevalence of halitosis among children studied according to Breath-Alert™, considering the organoleptic test as the gold standard.

Halitosis-Organoleptic test			No	Yes	Total
Halitosis-Breath-Alert™	Yes	Count	21	2	23
		% of total	14%	1.3%	15.3%
	No	Count	5	122	127
		% of total	3.3%	81.3%	84.7%
Total		Count	26	124	150
		% of total	17.3%	82.7%	100.0%

**Table 3:** Sensitivity, specificity, positive predictive value (PPV), and negative predictive value (NPV) of the Breath-Alert™ device as a diagnostic test for the detection of halitosis, considering the organoleptic test as the gold standard.

Sensitivity	Specificity	PPV	NPV
80.76%	98.38%	91.3%	96.06%
